# Local order and vibrational coupling of the C=O Stretching Mode of **γ**-Caprolactone in liquid binary mixtures

**DOI:** 10.1038/s41598-017-12030-1

**Published:** 2017-09-22

**Authors:** Wenwen Xu, Yanfang Sun, Xiaoping Dong, Si Li, Huigang Wang, Jiadan Xue, Xuming Zheng

**Affiliations:** 1 Department of Chemistry, Engineering Research Center for Eco-dyeing and Finishing of Textiles, MOE, Zhejiang Provincial Top Key Academic Discipline of Chemical Engineering and Technology, Zhejiang Sci-Tech University, Hangzhou, 310018 China; 2 College of Life Sciences, Zhejiang Sci-Tech University, Hangzhou, 310018 China

## Abstract

The isotropic and anisotropic parts Raman spectra of γ-Caprolactone in the binary mixture at different concentrations have been measured. The non-coincidence effect (NCE) of γ-Caprolactone was determined in carbon tetrachloride solution and DMSO solution. The NCE of the ν_11_(C=O) stretching mode in the γ-Caprolactone/DMSO mixtures exhibits a linear plot, in contrast to that in the γ-Caprolactone/CCl_4_ mixtures, which shows an upward (convex) curvature. The reduction and enhancement of the dimer structure (short-range orientational order) of γ-Caprolactone in the γ-Caprolactone/DMSO and γ-Caprolactone/CCl_4_ mixtures respectively may play a major role in shifting of peak frequencies, thus the geometries of monomer and dimer of γ-Caprolactone were calculated at the B3LYP-D3/6–311 G (d,p) level of theory. We proposed aggregated model to explain the γ-Caprolactone C=O vibration NCE phenomenon and its concentration effect and found it largely consistent with our experimental findings. Solvent dependent experiment show the value of NCE declined with the increase of the solvent dielectric constant under the same condition which is consistent with the Logan’s theory.

## Introduction

Intermolecular interactions play an important role in the fields of biochemistry, crystal engineering, supramolecular chemistry and physical organic chemistry^1–4^. Intermolecular interactions such as induced dipole interactions may determine what is the possible conformations of solute molecules in solvents^5,6^. Raman spectroscopic is an extraordinary method on the probe of the intermolecular interactions in liquid matter^7^. In addition, a variety of theoretic analysis methods have been proposed to explain the influence of solvent on the peak frequency shifts in binary liquid systems. For instance, the NCE of C=O and S=O stretching modes in the binary liquids have been reported by experiment or Monte Carlo (MC) or molecular dynamic (MD) simulation^8^ and explained by Logan’s model or Mirones’s model^9^. The purely dipolar model given by Logan describes the dependence of the noncoincidence splitting NCE value(Δν_NCE_) on environmental temperature (T), mole fraction (X_A_) and density (ρ) of reference substance, while the macroscopic continuum model of Mirone and Fini for the noncoincidence splitting of polar molecules depends linearly on the ratio of the volume fraction and the static dielectric constants of the solute and the solvent. In the model of Mirone and Fini, there is the existence of a threshold volume fraction below which the value of noncoincidence splitting becomes zero. These models with the results of theoretical calculation to elucidate the experimental phenomena are controversial. A justification for using the two models in the weak interaction systems will be provided later while discussing the results of NCE, especially the variation with volume fraction of the reference molecule γ-Caprolactone.

Raman spectroscopy has been widely used in studying the molecular interactions in terms of their vibrational frequency shifts disturbed by environmental changes^10^, such as hydrogen bond of C=O vibrations, dipole-dipole coupling of S=O, C=S, and amide group etc^11–14^. An example is the amide I region, dominated by signals of the backbone C=O vibrations, that encodes the secondary structure of a protein^15^. The frequencies of the vibration depends on the strength and polarity of the vibrating bonds and are thus influenced by intra- and intermolecular interactions, vibrational spectroscopy is exceptionally sensitive to changes in bond strength since a change of 0.02% can be easily detected^16^. In binary liquid mixtures, the vibrational frequencies shift of both the isotropic(ν_iso_) and anisotropic(ν_aniso_) Raman components of the reference vibrational mode with the change of concentration provide useful information about solute–solvent interaction and intermolecular forces^17–19^. This kind of phenomenon is known as Raman noncoincidence effect (NCE), the NCE value(Δν_nce_) is defined as Δν_nce_ = ν_aniso_ − ν_iso_^20–22^. It arises due to the manifestation of vibrational energy transfer induced by td–td interaction occurring in the presence of a short range or long-range orientational order of molecular dipoles^21,23^. The NCE is particularly significant in liquids structured by strong dipole-dipole interactions or hydrogen bonding^24^.

We have reported firstly the NCE of C=S stretching mode in ethylene trithiocarbonate^25^. Based on the matrix isolated Raman spectra, absolute Raman cross section character and DFT calculations, aggregated model has been proposed for the explanations of the C=O vibration NCE phenomenon^25–27^. This model has been successfully applied to explain NCE phenomenon.

γ-Caprolactone is a simple ring ester and a very common structural element in organic compounds, present in a variety of natural products such as pineapple and peaches. It is an important lactone natural perfume widely used as fragrance and food flavor, but they are also found as part of more complex frameworks, especially in drug-delivery systems^28^. They display a broad biological profile including strong antibiotic, antihelmitic, antifungal, antitumor, antiviral, antiinflammatory and cytostatic properties^29^, which makes them interesting lead structures for new drugs. On the other hand, the lactone unit represents a reactive functionality itself, being also a possible target for nucleophilic and electrophilic centers of biomolecules^30^. In many cases, the C=O stretching mode of the lactone ring are engaged in dynamic transition and molecular association. The choice of γ-Caprolactone was guided by the presence of the C=O group in its molecular moiety, suitable for studying the local order and vibrational coupling through the NCE of its vibrational band (C=O). The NCE of γ-Caprolactone may help us to probe the orientational organization of liquid matter and molecular self- association. Herein, the Raman spectroscopic noncoincidence effect of the C=O band of the γ-Caprolactone diluted in carbon tetrachloride has been measured at different concentrations. Density functional (DFT) calculations and the polarizable continuum model (PCM) were used to study the vibration frequency of γ-Caprolactone in CCl_4_, and its dimer at the hybrid B3LYP-D3 levels of theory with the 6–311 G(d,p) basis set by using the Gaussian 09 program^31^. The solvent polarity influence was included using the PCM, and the optimized geometry and the corresponding vibrational frequencies were obtained to verify the reasonability of dimer structure. Experimental results were satisfactorily agreed with the results obtained by DFT calculations. These studies are expected to be helpful in understanding the NCE phenomenon and concentration dependant of C=O stretching vibration in other ketones, and the aggregated model also help to comparatively understand some abnormal physical constant.

## Experimental and Computational Methods

Raman spectra were recorded for γ-Caprolactone (C_6_H_10_O_2_, 99% purity) at various solute concentration (volume fraction) ranging from 2.5% to 100% in Carbon tetrachloride solution (AR, Hangzhou Gaojing Fine Chemical Industry Co., Ltd). The 488 nm Raman spectra were obtained with the use of an experimental apparatus consisting of a triple monochromator (TriVista TR557, Princeton Instruments) equipped with an argon ion laser (Coherent, CVI MELLES GRIOT) as a source of exciting light at 488 nm (20 mW on the sample) and with a liquid nitrogen cooled CCD array (manufacturer, Princeton Instruments Inc.; model ID:LN/2048 × 512.B/I,UVAR.) allowing a wavenumber coverage of 1089 cm^−1^ and a spectral resolution (the instrumental apparatus function, FWHM) of 2.5 cm^−1^. The accuracy in the measurement of the band positions was 0.5 cm^−1^. The laser beam propagating orthogonally to the sample cell (along the *X* direction in the laboratory frame) was polarized perpendicular to the spectrometer’s optical axis and was focused on the sample with the use of a 60 × /0.42 f = 200 objective (S Plan APO HL), which, at the same time, collected the Raman-scattered light in a backscattering geometry. The polarization measurements were carried out in the VV and VH polarization configurations by vertically (V) polarizing the exciting laser light and by alternatively selecting the vertically (V) and horizontally (H) polarized components of the Raman scattered light with the use of a polarization sheet. The polarization measurements were calibrated by checking the depolarization factors of the bands of CCl_4_ at 314 cm^−1^ and 459 cm^−1^. The Raman measurements were performed at room temperature (293 K) and atmospheric pressurein all the runs. The spectral intensity of each mixture was constantly checked against that of the neat liquid normal species and, whenever necessary, the integration time was slightly varied to recover the same initial intensity condition of the latter.

The Fourier transform (FT)-Raman and FT-IR spectra were obtained with 2 cm^−1^ resolution using a FT-Raman spectrometer at 1064 nm excitation (Thermo Nicolet 960, Thermo Fisher Nicolet, USA) and a FT-IR spectrometer (Thermo Nicolet avatar 370, Thermo Fisher Nicolet, USA).

We converted Raman intensity from scattering activities using the Multiwfn software (http://multiwfn.codeplex.com/releases). Multiwfn is an extremely powerful wavefunction analysis program, supports almost all of the most important wavefunction analysis methods^32^. In our case, the excitation frequency is 20491.80 cm^−1^ (corresponding to 488 nm) and the conversion relationship is shown as^33^:

$${I}_{i}=\frac{C{({\nu }_{0}-{\nu }_{i})}^{4}{S}_{i}}{{\nu }_{i}[1-\exp (-\frac{hc{\nu }_{i}}{kT})]}$$

where *S*_*i*_ is the Raman activity, *I*_*i*_ is the Raman intensity, *ν*_*o*_ is the exciting frequency in reciprocal centimeters, *ν*_*i*_ is the vibrational frequency of the *i*th normal mode, *h*, *c*, and *k* are universal constants, and C is a suitably chosen common normalization factor for all peak intensities.

Computational chemical methods help to better understand the photophysical and photochemical characteristics of the molecule, Herein, Density functional theory (DFT) calculations were carried out using the hybrid B3LYP-D3 function to determine the optimized geometry and vibrational frequencies. Complete geometry optimization were computed by using the B3LYP-D3/6–311 + G (d,p) level of theory for the ground state of γ-Caprolactone. All of the DFT calculations made use of the Gaussian program software suite^31^.

## Results and Discussion

**Figure 1:**
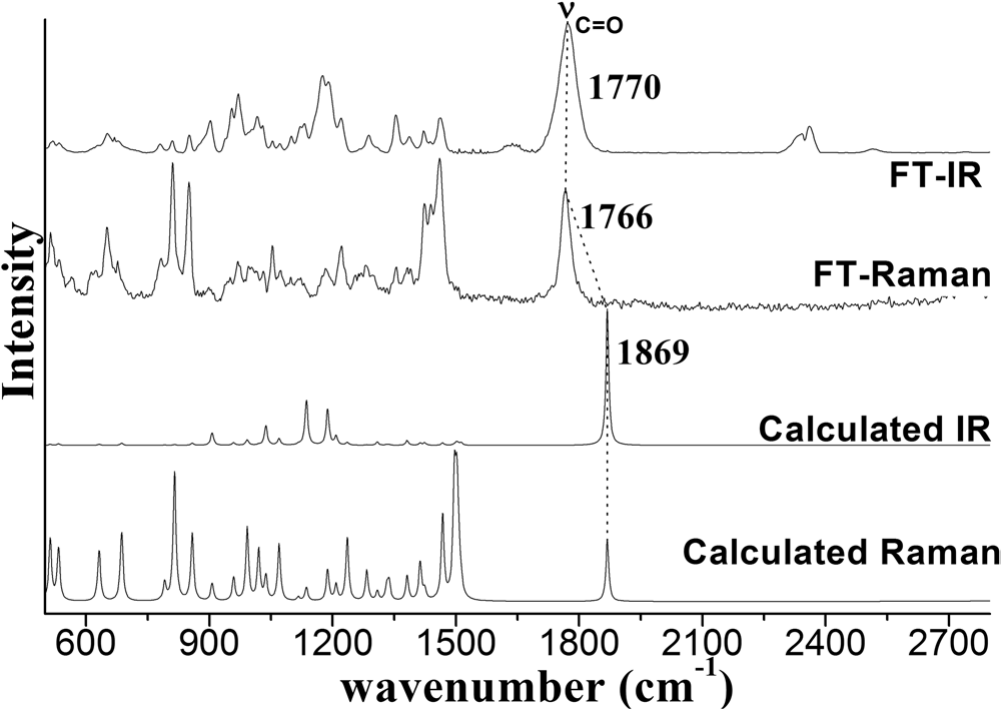
Comparison of the FT-IR and FT-Raman with calculated Raman and IR spectra of γ-Caprolactone.

We have carried out DFT calculations for γ-Caprolactone in order to help elucidate the vibrational bands observed in the experimental FT-Raman and FT-IR spectra of γ-Caprolactone. Figure 1 displays the comparison of the calculated Raman and IR spectra of γ-Caprolactone with the FT-Raman spectrum and FT-IR spectrum of γ-Caprolactone. Table 1 lists a comparison of the B3LYP-D3/6–311 G(d,p) calculated vibrational wavenumber with experimental FT-Raman and FT-IR values. The overall agreement between the linear regression scaled DFT calculated vibrational wavenumber and the experimental values is good for γ-Caprolactone. The dashed lines in Fig. 1 indicate the correlation of the vibrational modes of γ-Caprolactone in calculated Raman and IR spectrum to those corresponding fundamental modes of γ-Caprolactone in FT-Raman spectrum and FT-IR spectrum respectively. The 1844 cm^−1^ band is assigned to the C=O stretch. Its frequency difference between the FT-Raman spectrum and FT-IR spectrum is noticeable (4 cm^−1^ frequency difference). This frequency difference is one key character for noncoincidence effect, other experiment proofs including the isotropic and anisotropic Raman spectra at different concentration will be present later. Primary data demonstrates that the γ-Caprolactone may exist a short or long-range orientational order which is induced by the C=O vibrational td–td interaction.

**Table 1:** B3LYP-D3/6–311 G(d,p) computed frequency, depolarization ration, ZPE corrected total energy of γ-Caprolactone monomer and dimer in carbon tetrachloride Solution.

modes	Computed/cm^−1^				Experiment/cm^−1^		descriptions
	mono	dimer			Raman	IR	
	Freq.	D. ratio	Freq.	D. ratio			
ν_1_	3124	0.55	3129/3126	0.61/0.64			H-C-H asymmetric stretch
ν_2_	3112	0.31	3119/3116	0.37/0.20	2983vs	2970s	H-C-H asymmetric stretch
ν_3_	3100	0.49	3101/3099	0.56/0.53			-CH_3_ in-plane-bend
ν_4_	3091	0.75	3098/3093	0.50/0.69			H-C-H asymmetric stretch
ν_5_	3066	0.66	3080/3075	0.23/0.75			H-C-H asymmetric stretch
ν_6_	3048	0.63	3068/3059	0.12/0.74			H-C-H symmetric stretch
ν_7_	3043	0.08	3058/3044	0.11/0.16			H-C-H symmetric stretch
ν_8_	3032	0.28	3040/3039	0.13/0.16			H-C-H symmetric stretch
ν_9_	3025	0.01	3027/3025	0.03/0.05	2939vs	2941s	-CH_3_ umbrella
ν_10_	3012	0.26	3023/3015	0.03/0.37	2885vs	2883s	C-H symmetric stretch
**ν**_**11**_	**1844**	**0.51**	**1824/1807**	**0.68/0.32**	**1766s**	**1770vs**	**C=O stretch**
ν_12_	1510	0.74	1516/1510	0.56/0.75			H-C-H scissor
ν_13_	1502	0.75	1506/1505	0.73/0.75			H-C-H scissor
ν_14_	1498	0.75	1501/1500	0.75/0.73			H-C-H scissor
ν_15_	1492	0.72	1496/1493	0.73/0.71	1460s	1460w	H-C-H scissor
ν_16_	1462	0.71	1465/1457	0.75/0.70	1423	1421w	H-C-H scissor
ν_17_	1421	0.46	1425/1422	0.66/0.59			C-H out-of-plane bend
ν_18_	1412	0.73	1418/1416	0.72/0.70			C-H in-plane-bend
ν_19_	1381	0.74	1397/1387	0.66/0.75			C-H out-of-plane bend
ν_20_	1338	0.74	1353/1347	0.69/0.68			H-C-H wag
ν_21_	1333	0.74	1340/1337	0.74/0.72			H-C-H wag
ν_22_	1311	0.53	1318/1316	0.71/0.72			H-C-H wag
ν_23_	1283	0.73	1291/1287	0.70/0.71			H-C-H twist
ν_24_	1237	0.73	1248/1240	0.71/0.75	1223	1221	C-H in-plane-bend
ν_25_	1208	0.75	1216/1213	0.74/0.73			H-C-H twist
ν_26_	1189	0.62	1199/1194	0.73/0.31			C-C stretch
ν_27_	1139	0.47	1152/1144	0.71/0.39			C-C stretch
ν_28_	1118	0.75	1128/1126	0.73/0.74			H-C-H twist
ν_29_	1067	0.41	1067/1066	0.58/0.31			C-C stretch
ν_30_	1032	0.58	1027/1025	0.50/0.70			C-O stretch
ν_31_	1020	0.39	1024/1024	0.71/0.20			H-C-H rock
ν_32_	990	0.27	990/989	0.21/0.21			C-C stretch + C-O stretch
ν_33_	959	0.67	962/960	0.67/0.71			C-C stretch
ν_34_	909	0.55	917/914	0.72/0.59			C-O stretch
ν_35_	859	0.17	865/862	0.13/0.19	850	850	C-C stretch + H-C-H rock
ν_36_	816	0.10	820/818	0.18/0.08	810	810	C-C stretch + H-C-H rock
ν_37_	792	0.16	800/795	0.21/0.10			H-C-H rock
ν_38_	687	0.46	692/689	0.48/0.41	650	652	C-C-O scissor
ν_39_	633	0.75	642/635	0.75/0.74			C-C-O scissor
ν_40_	533	0.65	547/538	0.62/0.75	513	519	H-C-H rock
ν_41_	512	0.11	515/514	0.11/0.15	440		C=O in-plane-bend
ν_42_	458	0.14	466/460	0.31/0.14	393		H-C-H rock
ν_43_	364	0.42	367/367	0.65/0.34			C-C-C scissor
ν_44_	247	0.39	256/256	0.65/0.29			-CH_3_ rock
ν_45_	215	0.53	227/218	0.73/0.57			-CH_3_ rock
ν_46_	155	0.69	181/168	0.74/0.54			H-C-H rock
ν_47_	122	0.74	141/131	0.65/0.74			H-C-H rock
ν_48_	85	0.75	127/101	0.75/0.71			C-C in-plane-bend
ν_49_			88/77	0.64/0.71			Relative rotation
ν_50_			66/55	0.67/0.59			Relative rotation
ν_51_			30/15	0.72/0.75			Relative translation
ZPE Corrected Total Energy(a.u.)			Monomer	HF = −1162340.74			
			Dimer	HF = 2324732.54	ΔE = HF(Dimer)−2HF(Monomer) = −51.06		

In dimer, there are in-phase and out-of-phase vibrational modes, in-phase vibrational frequency below than out-of-phase vibrational frequency.

**Figure 2:**
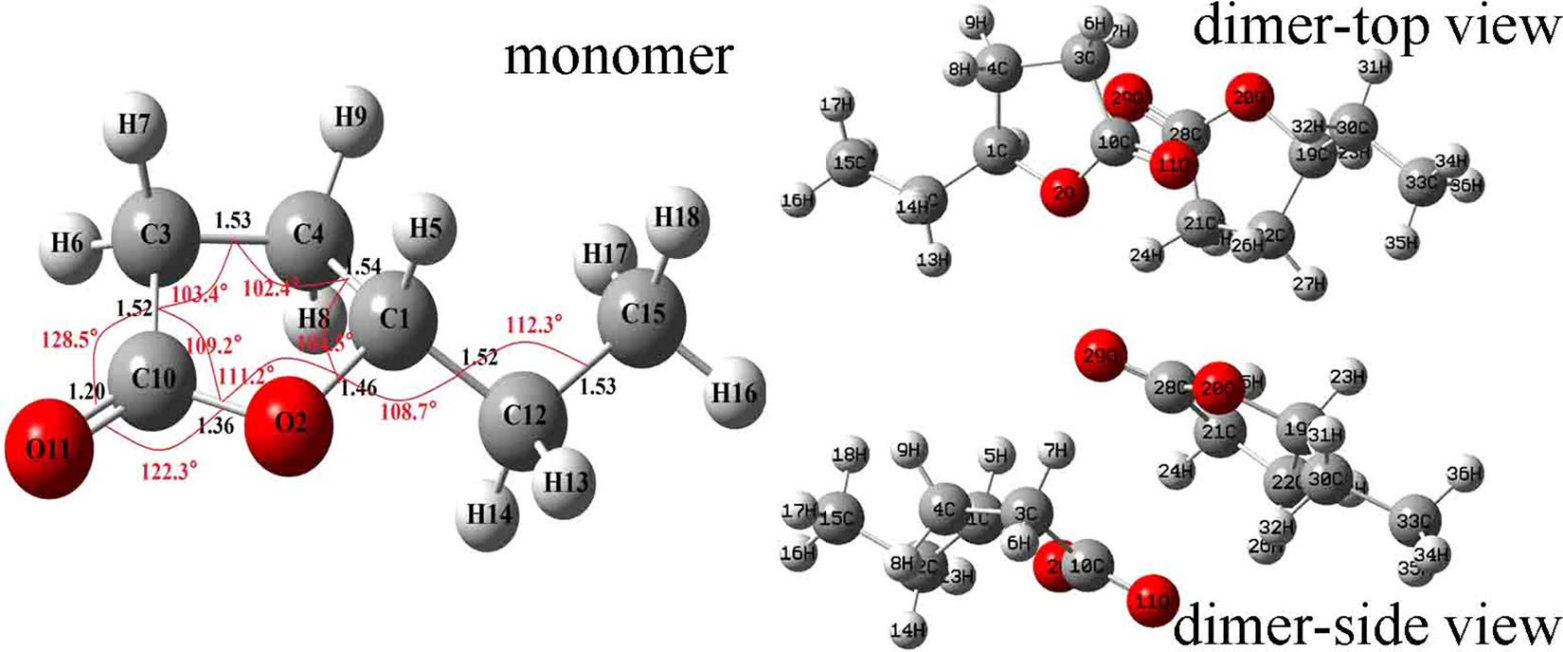
B3LYP-D3/6–311 G(d,p) computed geometry parameters of γ-Caprolactone and its aggregates.

We performed full geometry optimization of the γ-Caprolactone. In order to establish the most stable conformation as the initial point for further calculations, the molecule was submitted to a rigorous conformational analysis around all bonds having free rotation. We also calculated γ-Caprolactone monomer and its dimer using the PCM solvent model in CCl_4_^34^ Solution at the B3LYP-D3/6–311 G (d,p) level of theory, no imaginary vibrational frequencies were found in the further vibration calculation. The most stable geometries of the monomer and dimer are shown in Fig. 2, the γ-Caprolactone assembled in a face to face, head to tail antiparallel dimeric form through strong intermolecular interaction. All DFT calculated vibrational frequencies, depolarization ratio and ZPE Corrected Total Energy for γ-Caprolactone monomer and its dimer are given in Table 1. By intermolecular interaction, molecules can form dimers. Dipole-dipole interactions tend to align the molecules to reduce potential energy and increase attraction. Table 1 and Fig. 2 show that, the coupling of two C=O stretching in the dimer differentiates the interaction in two extreme ways. One way of the C=O stretching interaction is in-phase, the second way is out-of-phase, which leads to C=O vibrational frequency and depolarization ratio discrepancy. In-phase C=O stretching frequency lies below than out-of-phase one.

**Figure 3:**
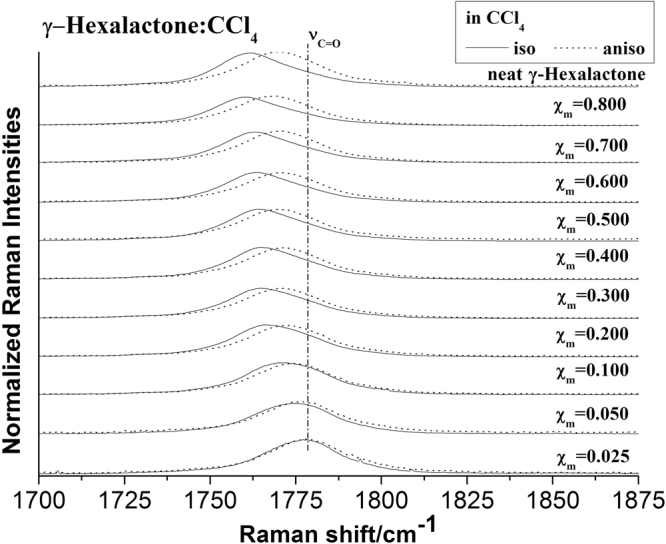
The ν_11_(C=O) vibration isotropic and anisotropic parts of the Raman spectra in the region 1770–1875 cm^−1^ for γ-Caprolactone and ten other volume fractions of γ-Caprolactone, 0.800, 0.700, 0.600, 0.500, 0.400, 0.300, 0.200, 0.100, 0.050 and 0.025 in the binary mixture (C_6_H_10_O_2_ + CCl_4_).

The 36 atoms of γ-Caprolactone dimer give rise to 102 normal modes of vibration. The overall 102 normal modes of vibration of this dimer may be considered to be comprised of 96 normal modes arising from these two γ-Caprolactone molecules in-phase and out-of-phase coupling, six modes associated with the relative translation and rotation of two γ-Caprolactone molecules. Detail description and the comparison of calculated frequencies and corresponding depolarization ratio for monomer and dimer structures, as well as experimental Raman and IR frequencies are listed in Table 1. Corresponding in-phase and out-of-phase vibration modes may differ in wavenumbers and depolarization ratio, and the magnitude of these splitting will depend on the strength of interaction between different parts of the neighbor molecules. Table 1 show that although the aggregated structure of the coupling removes the degeneracy of the C=O stretching vibrational level, only few pairs split prominently, but still beyond the resolution limits for Raman instrument, thus forms a strong broad peak. Thanks to the significant difference in depolarization ratio of C=O stretching pairs, it make possible to collect preferential parallel or perpendicular polarized component Raman spectra, corresponding to isotropic or anisotropic parts, by selecting polarization sheet with VV and VH polarization configurations. This parallel or perpendicular polarized spectra preferential collect one component of the C=O stretching pairs, thus separate these two components and lead to the observation of NCE phenomenon. In brief, only those pairs that with prominent vibrational frequency difference and depolarization ratio difference have NCE effect. Examining the DFT calculated frequency in Table 1, only ν_11_(C=O) stretching conform to these rules. The experimental Raman spectra and the NCE phenomenon of γ-Caprolactone also prove this calculation. Figure 3 shows the isotropic and anisotropic Raman spectrum of neat γ-Caprolactone for ν_11_(C=O) stretching mode. The isotropic peak frequencies at 1761.5 cm^−1^ and anisotropic peak at 1771.0 cm^−1^ were assigned to the calculated frequency at 1807 cm^−1^ and 1824 cm^−1^ respectively, the corresponding depolarization ratio are 0.32 and 0.68. The calculations on dimer model are in good agreement with the experiment non-coincident isotropic and anisotropic Raman data.

**Figure 4:**
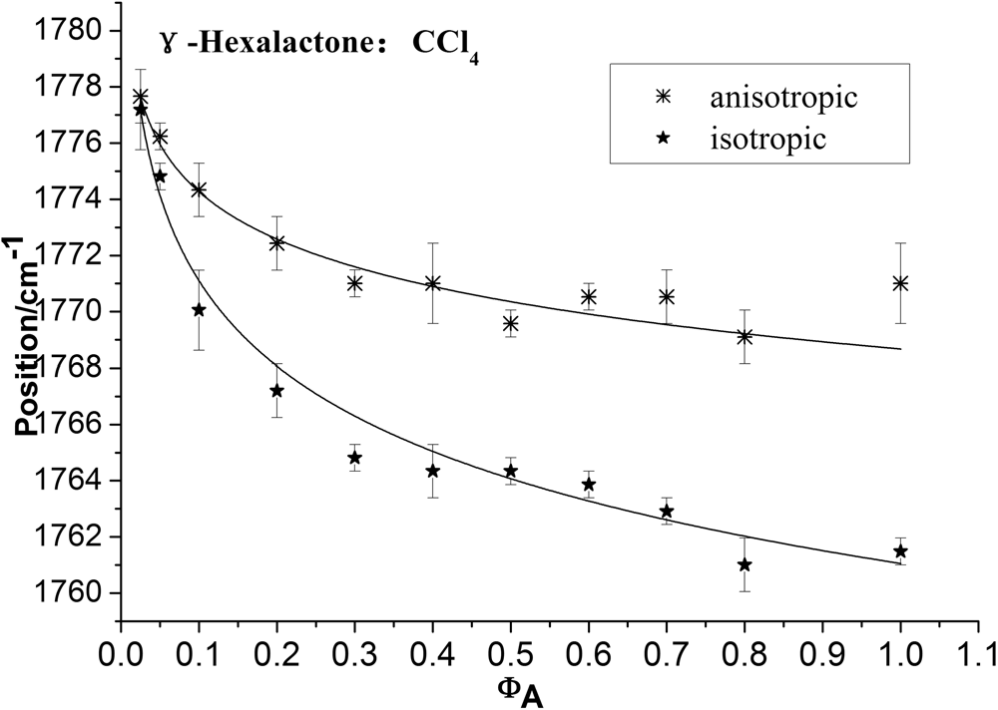
Variation of isotropic and anisotropic Raman peak frequencies of C=O stretching mode of γ-Caprolactone as a function of solute volume fractions (C_6_H_10_O_2_ + CCl_4_). The curves were fitted with negative exponential functions [anisotropic: *ƒ*(*x*) = 9.11*e* ^−7.27*x*^ + 1769.96, *r* ^2^ = 0.9734; isotropic: *ƒ*(*x*) = 16.26*e* ^−5.65*x*^ + 1762.42, *r* ^2^ = 0.9899].

**Figure 5:**
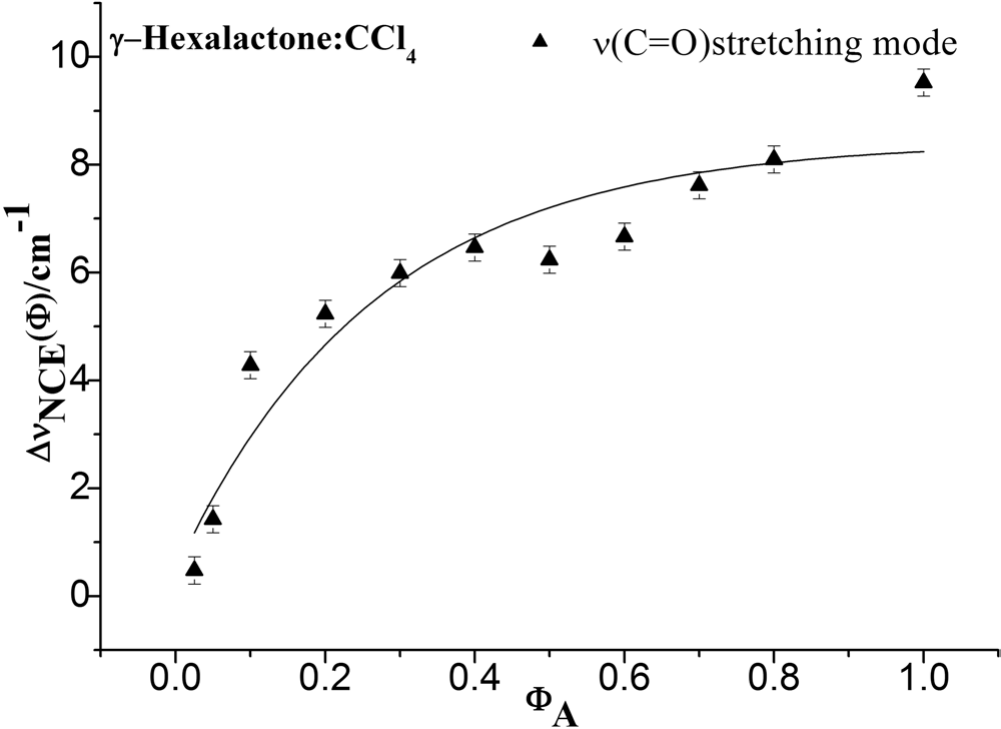
Variation of NCE of C=O stretching mode of γ-Caprolactone as a function of solute volume fractions (C_6_H_10_O_2_ + CCl_4_). The curve were fitted with exponentialfunction [*ƒ*(*x*) = 8.43–7.97*e* ^−3.74*x*^, *r* ^2^ = 0.9148].

To characterize the concentration effect, Fig. 3 also lists the isotropic and anisotropic spectra at a variety of volume fractions of γ-Caprolactone in CCl_4_. It demonstrates that both the isotropic and anisotropic Raman frequency of C=O stretch increase in wavenumber with the decrease of γ-Caprolactone concentration, the separation between isotropic and anisotropic Raman frequency is 9.5 cm^−1^ in neat γ-Caprolactone while disappears at χ_m_ = 0.025. The FWHM (full width at half maxima) of the C=O stretching modes also get smaller and the peak get sharp with the decrease solute concentrations. To scrutinize the rule of Raman frequency changes with volume fraction, the peaks frequency corresponding to the C=O stretching modes for the isotropic (I_iso_) and the anisotropic (I_aniso_) parts of the Raman spectra in neat γ-Caprolactone as well as in ten other volume fractions, 0.800, 0.700, 0.600, 0.500, 0.400, 0.300, 0.200, 0.100, 0.050 and 0.025 in the binary mixture (C_6_H_10_O_2_ + CCl_4_) is drawn from Fig. 3. It can be roughly understood from the experimentally measured Raman peak frequencies of isotropic (ν_iso_) and anisotropic (ν_aniso_) components of C=O stretching mode plotted as a function of volume fractions in CCl_4_ in Fig. 4. The Δυ_NCE_ becomes zero at a threshold concentrationφ_A_, which depends on the nature of the solvent. The Raman peak frequencies of both components show an increase in wavenumber with the decrease of solute concentrations. The isotropic ν_11_(C=O) frequencies for neat γ-Caprolactone and volume fractions of 0.025 γ-Caprolactone are 1761.5 cm^−1^ and 1777.2 cm^−1^ respectively. That is to say, from the highest to the lowest concentration of γ-Caprolactone, the wavenumbers of C=O stretching blue shifted about 15.7 cm^−1^, while of other vibrational bands did not shift. Meanwhile the value of Δυ_NCE_ = ν_aniso_ − ν_iso_ was calculated and was plotted with volume fractions in CCl_4_ in Fig. 5, it goes on decreasing upon dilution with CCl_4_ from 9.5 cm^−1^ in neat γ-Caprolactone and reduces to the quite low values of 0.48 cm^−1^ at χ_m_(γ-Caprolactone in CCl_4_) = 0.025 for the C=O stretching modes. The large change (9.02 cm^−1^) from neat to infinite dilution is due to the decrease in td–td interaction.

The noncoincidence between isotropic and anisotropic spectra of γ-Caprolactone implies that there may be chances of formation orientational order by dipoles coupling. Correspondingly, dipole-dipole interactions tend to align the molecules to reduce potential energy and increase attraction. According to the energy difference between the dimer and monomer in Table 1 (51.06 kJ/mol energy difference), γ-Caprolactone molecules are more likely to form dimer. At high dilution the solvent molecules diffuse towards the reference molecules and break its association ordered structure, there-by weakening the dipole–dipole interaction of the solute molecules. Compared with the result calculation, during the dilution process the solute-solvent interaction breaks the dimer to monomer. The energy difference between monomer and dimer is 51.06 kJ/mol. It is easy to be overcome and transformed between each other. With the decrease of γ-Caprolactone concentrations the dimer gradually breaks into monomer and the Raman spectra gradually transform from dimer character to monomer character. The DFT calculated frequencies in Table 1 shows that among all the vibration modes only ν_11_(C=O) has prominent vibrational frequency difference, what is more it also gives that the ν_11_ frequency of monomer (1844 cm^−1^) is higher than that of dimer (1824/1807 cm^−1^). Therefore, the DFT calculation manifested the phenomenon that we observed in Fig. 3, i.e., the C=O stretching red shift and the peak get sharpened and symmetric upon dilution. The NCE get smaller and disappear in extremely diluted solution.

The NCE may be both positive and negative depending upon the orientation of the dipoles during their interactions. In this study, the NCE of γ-Caprolactone is positive which may be due to antiparallel side-by-side interaction of the intermolecular dipoles, similar to the calculated geometry of the dimer shown in Fig. 2. It has been widely known that the NCE represents a spectroscopic manifestation of the occurrence of the resonant intermolecular interaction between nearby IR-active oscillators through the td–td interaction mechanism^14,35^. This interaction induces resonant vibrational energy transfer occurring in the presence of a short-range orientational order by dipolar or nondipolar forces^6,14,36^. The NCE is related to both the degree of short-range order and to the strength of the intermolecular vibrational coupling. Within the Born-Oppenheimer approximation, the electric dipole moment should stay constant with different concentration. By contrast, the dilution of the solute alters the short-range order of C=O stretching normal coordinate and their relative alignment distributions of the interacting dipoles. This result in weakening of the pair interaction which is formed due to cage effect where solvent molecules surround the solute molecule, and leads to decrease in non-coincidence effect on further dilution of solute.

To explore the NCE behavior in solvents of lower and higher polarity, we extend this subject to polar solvents DMSO and make a complete comparison between the experimental results.

**Figure 6:**
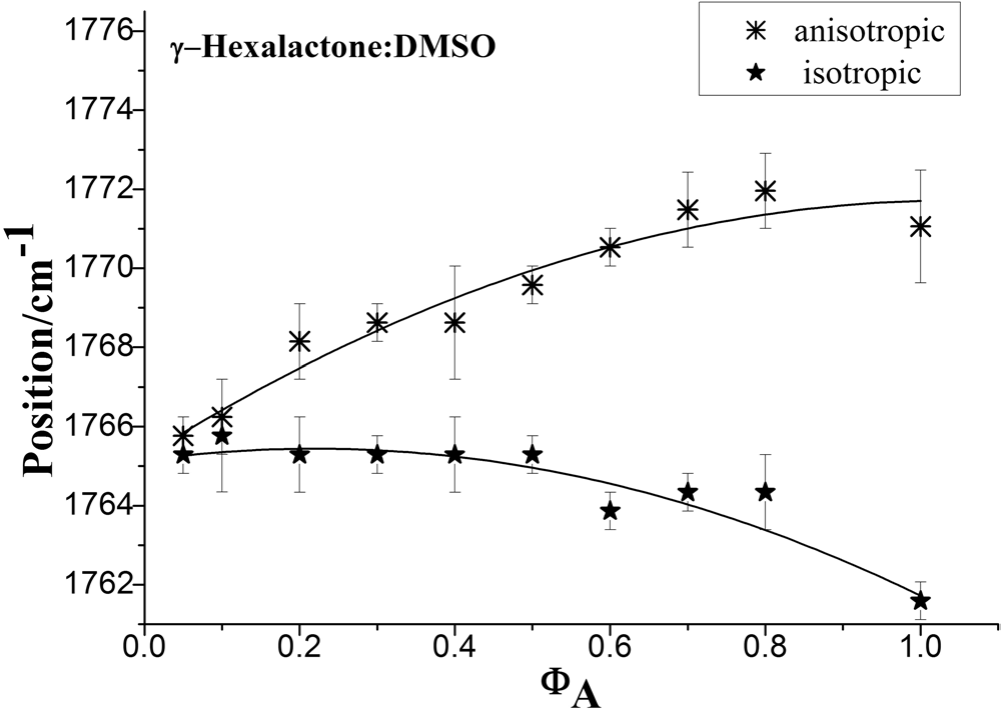
Concentration dependence of the isotropic and anisotropic Raman frequency for the ν_11_(C=O) stretching mode of γ-Caprolactone in the binary mixture (C_6_H_10_O_2_ + DMSO). The curves were fitted with exponential functions [anisotropic: *ƒ*(*x*) = 1773.37–8.26*e* ^−1.75*x*^, *r* ^2^ = 0.9719; isotropic: *ƒ*(*x*) = 1765.34–0.02*e* ^5.20*x*^, *r* ^2^ = 0.9678].

We also report the concentration dependence of the isotropic and anisotropic Raman frequency of the ν_11_(C=O) mode of γ-Caprolactone in γ-Caprolactone /DMSO mixtures. The experimentally measured ν_iso_ and ν_aniso_ components of C=O stretching mode plotted as a function of volume fractions in DMSO in Fig. 6. It illustrates that the isotropic Raman frequency increase in wavenumber with the decrease of γ-Caprolactone concentrations in DMSO, whereas the anisotropic component decrease with the dilution of γ-Caprolactone. The fitting curve displays a nonlinear concentration dependence with a upward (convex) curvature with concentration, rather than the downward (concave) curvature observed in nonpolar solvents such as CCl_4_ shown in Fig. 4.

**Figure 7:**
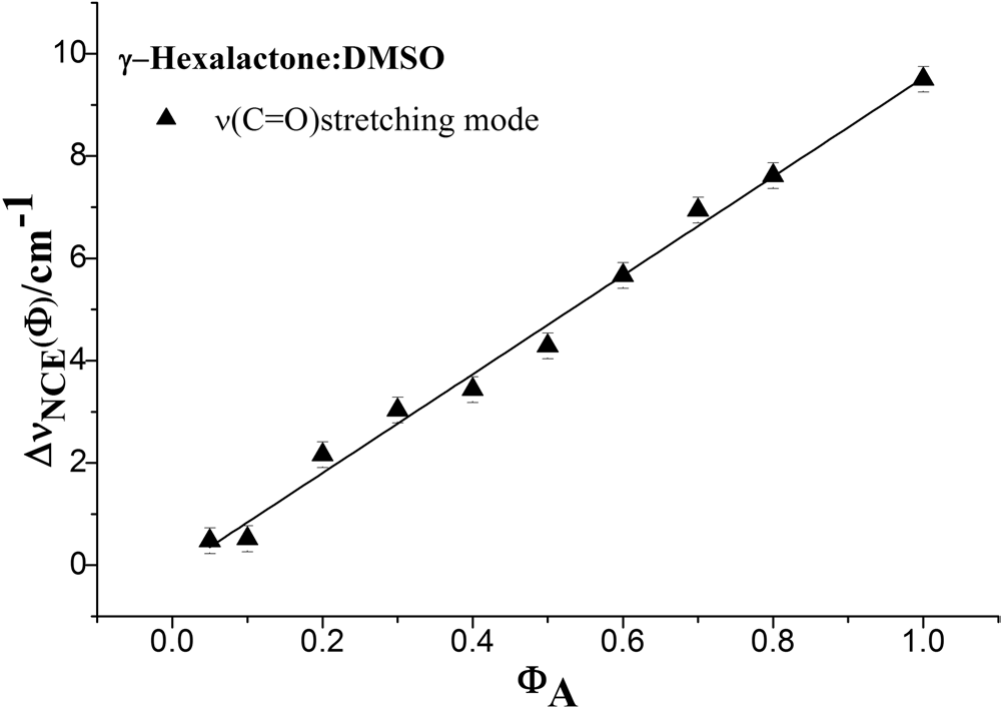
Concentration dependence of the NCE of C=O stretching mode of γ-Caprolactone in the binary mixture (C_6_H_10_O_2_ + DMSO). The data were fitted by a linear curve [*ƒ*(*x*) = 9.65*x*−0.13, *r* ^2^ = 0.9908].

The values of NCE calculated according to Fig. 6 are shown in Fig. 7. It is clearly seen in Fig. 7 that the experimental concentration dependence of NCE presents linear plot. It indicates that DMSO molecules, by virtue of having a dipole moment larger than that of γ-Caprolactone, reduce the dimer structure (short-range orientational order) of γ-Caprolactone in the mixtures. This behavior is in contrast to that observed for the NCE in the γ-Caprolactone/CCl_4_ mixtures. As shown in Fig. 4, we can see the downward (concave) curvature for the concentration dependence of the NCE in this case. The nonpolar nature of the CCl_4_ molecules reinforces the dimer structure (short-range orientational order) of γ-Caprolactone in the mixtures.

**Figure 8:**
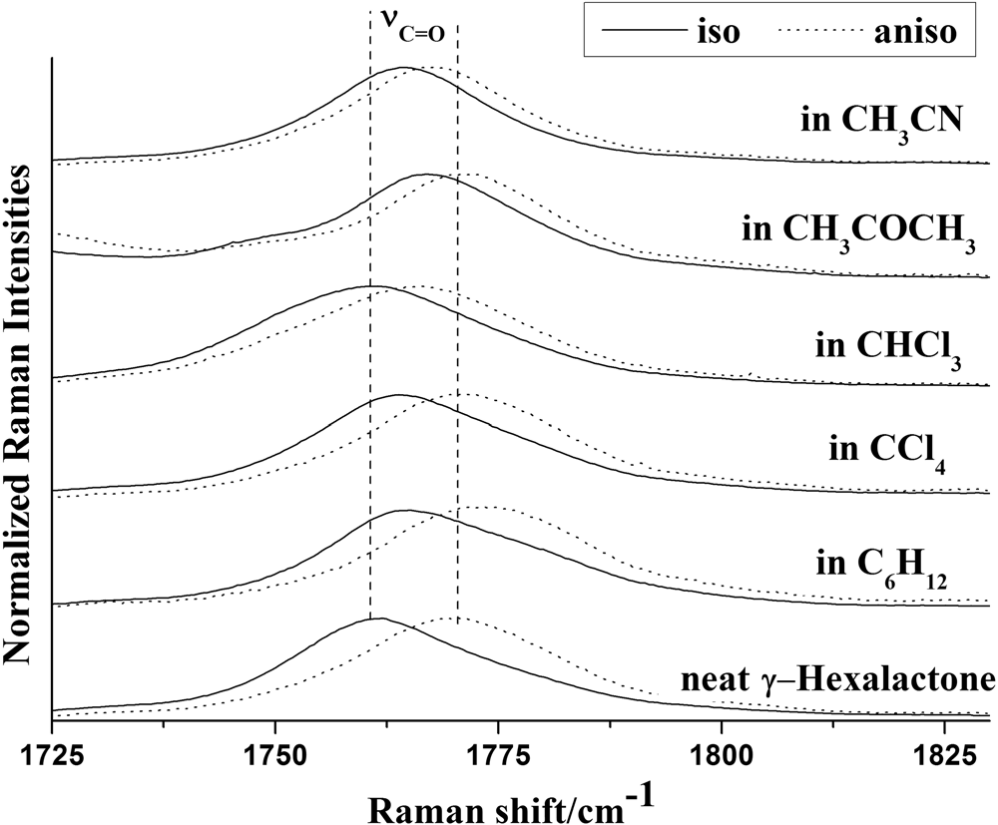
The isotropic and anisotropic parts of the ν_11_(C=O) vibration Raman spectra of γ-Caprolactone in the binary mixture with different solvents (ϕ_A_ = 0.500).

**Figure 9:**
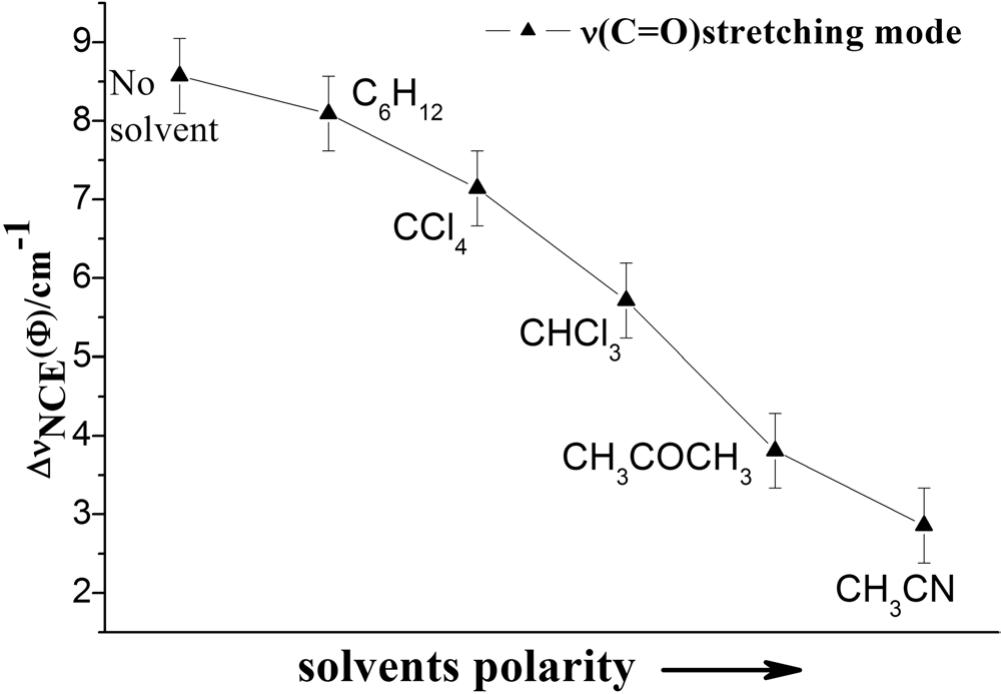
Variation of NCE of C=O stretching mode of γ-Caprolactone as a function of solvents dielectric constant.

The comparison between Fig. 5 and Fig. 7 demonstrates that the NCE character is largely determined by dipole moment of solvent. To study the influence of dipole moment of the solvent upon NCE, we collected the isotropic and anisotropic Raman spectra of γ-Caprolactone in a series of solvent with different static dielectric constant which was shown in Fig. 8. The corresponding NCE were calculated and illustrated in Fig. 9. Generally the value of NCE declined with the increase of the solvent dielectric constant with the same concentration. This rule is in consistency with the Logan’s theory^37^.

To investigate the NCE solvent polarity dependent properties and demonstrate the rationality of dimer model, Density functional (DFT) calculations and the polarizable continuum model (PCM) were applied to its dimer structure at the hybrid B3LYP-D3 levels of theory with the 6–311 G(d,p) basis set by using the Gaussian 09 program. The solvent polarity influence was calculated using the PCM, and the optimized geometry and the corresponding vibrational frequencies were obtained to verify the reasonability of dimer structure. Table 2 shows the DFT/PCM calculated C=O vibrational frequencies, depolarization ratios, intermolecular distance (R_d_/Å), NCE and ΔE (energy difference relative to monomer) in a variety of solvents. With the decrease of solvent dielectric constant, the two monomers of γ-Caprolactone dimer became closer, and the value of NCE increases while the energy difference relative to monomer ΔE decreases. These results are consistent with the experiments results shown in Fig. 9. The value of ΔE again verifies that the stronger polar solvent will weaken the dimer structure of γ-Caprolactone in the mixtures while the nonpolar solvent can reinforce the dimer structure.

**Table 2:** The DFT/PCM calculated C=O vibrational frequencies, depolarization ratios, intermolecular distance (R_d_/Å), NCE and ΔE of γ-Caprolactone dimer in in a variety of solvents.

solvents	Dielectric constant (ε)	Dimer		R_d_/Å	NCE/_cm_^−1^	ΔE/kJ/mol
		Freq.	D. ratio			
CH_3_CN	35.69	1803/1792	0.66/0.32	3.30247	11	−37.38
CH_3_COCH_3_	20.70	1804/1793	0.66/0.32	3.29985	11	−38.14
CHCl_3_	4.18	1814/1800	0.66/0.32	3.28031	14	−43.85
CCl_4_	2.24	1824/1807	0.68/0.32	3.24945	17	−51.06
C_6_H_12_	2.02	1826/1809	0.69/0.32	3.24190	17	−52.33
γ-Caprolactone	1.58	1840/1818	0.66/0.30	3.18000	22	−63.21

All results that we have obtained, from experiments, theoretical dimer model, and computational calculation, demonstrate a consistent picture of the relation between the NCE behavior, a spectroscopic feature of vibrational Raman bands, and the effects of the dipolar interactions in liquid mixtures at molecular level.

## Conclusion

The Raman spectroscopic noncoincidence effect of the υ(C=O) band of the γ-Caprolactone in the binary mixture has been firstly reported and the Δυ_nce_ has been measured at different concentrations. We found that in aprotic solvents the shape of the graph of NCE vs concentration depends on the relative values of the dielectric constants: the slope increases with increasing concentration when the solvent has a higher dielectric constant than the solute, and decreases in the reverse case. The monomer and dimer of γ-Caprolactone were calculated at the B3LYP-D3/6–311 G (d,p) level of theory, which makes it easy and accurate to investigate the weak interaction changes between molecules such as concentration dependant and NCE properties. During the dilution process the solute-solvent interaction weaken the dipole-dipole interaction, which makes the Raman spectra gradually transformed from dimer character to monomer character thus blueshift the C=O stretching. These Density functional theory (DFT) calculations provided a satisfactory results and fit well with the experimental findings. Solvent dependent experiment shows the value of NCE declined with the increase of the solvent dielectric constant under the same condition.
